# Pooling of continuous features provides a unifying account of crowding

**DOI:** 10.1167/16.3.39

**Published:** 2016-02-26

**Authors:** Shaiyan Keshvari, Ruth Rosenholtz

**Affiliations:** shaiyan@mit.edu; rruth@mit.edu; Department of Brain and Cognitive Sciences, MIT, Cambridge, MA, USA; Computer Science and Artificial Intelligence Laboratory, Cambridge, MA, USA

**Keywords:** *crowding*, *computational modeling*, *peripheral vision*, *texture perception*

## Abstract

Visual crowding refers to phenomena in which the perception of a peripheral target is strongly affected by nearby flankers. Observers often report seeing the stimuli as “jumbled up,” or otherwise confuse the target with the flankers. Theories of visual crowding contend over which aspect of the stimulus gets confused in peripheral vision. Attempts to test these theories have led to seemingly conflicting results, with some experiments suggesting that the mechanism underlying crowding operates on unbound features like color or orientation (Parkes, Lund, Angelucci, Solomon, & Morgan, 2001), while others suggest it “jumbles up” more complex features, or even objects like letters (Korte, 1923). Many of these theories operate on discrete features of the display items, such as the orientation of each line or the identity of each item. By contrast, here we examine the predictions of the Texture Tiling Model, which operates on continuous feature measurements (Balas, Nakano, & Rosenholtz, 2009). We show that the main effects of three studies from the crowding literature are consistent with the predictions of Texture Tiling Model. This suggests that many of the stimulus-specific curiosities surrounding crowding are the inherent result of the informativeness of a rich set of image statistics for the particular tasks.

## Introduction

Colloquially, peripheral vision refers to vision outside of the center of gaze (i.e., outside the central 1°–2° of visual angle known as the fovea).^1^ Peripheral vision is where you are not looking (i.e., not pointing your eyes). Despite it being markedly worse than central vision, peripheral vision is a key player in many natural tasks, including gaze guidance, object recognition, scene perception, and navigation (Strasburger, Rentschler, & Jüttner, 2011; Whitney & Levi, 2011).

Peripheral vision has limited acuity and color perception compared with central vision (Anstis, 1998; Strasburger et al., 2011). Whereas these deficits are well studied and significant, they only explain a small fraction of the difficulty viewers have when performing peripheral tasks. For example, the modest decrease in acuity with eccentricity—distance to the point of fixation—would imply that one could read an entire page of 12-pt. text at reading distance without moving one's eyes (based on functions relating letter acuity to eccentricity from Anstis, 1998). The crucial limit to peripheral processing is not lack of acuity, but rather visual crowding.

Crowding refers to the empirical phenomena in which observers have difficulty performing a peripheral task, such as identifying or discriminating a target, when that target is surrounded by nearby flankers or is otherwise too complex or cluttered. Traditionally, crowding has often been demonstrated and studied with recognition of a target letter flanked by other letters, but crowding is fundamental to peripheral vision, and not specific to letter stimuli (Pelli & Tillman, 2008). A crowded stimulus often subjectively looks “mixed up,” such that the details and exact locations of the stimulus' visual features become difficult to discern. Lettvin (1976) described the subjective experience as one in which the stimulus “only seems to have a ‘statistical' existence… The loss of spatial order does not involve angles or any other property that we commonly discuss…” This line of thinking has developed into conceptualization of crowding as “forced texture perception.”

What, however, does “forced texture perception” mean? Researchers have answered this question in different ways, analogous to different dominant approaches to modeling texture perception (Rosenholtz, 2014). Whether attempting to predict texture segmentation, discrimination, or representation, most models of texture perception fall into two broad classes: models that describe texture in terms of the features of discrete, presegmented texture elements (“things”), and models that describe texture by a rich set of image statistics (“stuff”). As an example of the things class of models, some theories of texture segmentation represent texture by a set of discrete features, called textons. Whether segmentation occurs depends on whether two abutting textures contain the same number of attributes, like vertical lines, endstops, closed curves, and arrow junctions (Julesz, 1981; Julesz & Bergen, 1983; Pomerantz & Cragin, 2015). On the other hand, filter–nonlinearity–filter models of texture segmentation (Landy & Bergen, 1991; Malik & Perona, 1990; Rosenholtz, 2000), as well as texture representations based on image statistics (Heeger & Bergen, 1995; Portilla & Simoncelli, 2000), fall into the class of stuff models of texture perception.

If we characterize crowding models in this fashion, several prominent classes of models are thing models, operating on discrete features. In averaging models, the visual system is presumed to measure, say, the orientation of each individual item, and encode only a few summary statistics such as the mean and variance (Greenwood, Bex, & Dakin, 2012; Parkes et al., 2001). On the other hand, substitution models hypothesize that peripheral vision detects discrete things—for example, texton-like features or letter identities—but loses or jumbles their location information (Pelli, Cavanagh, Desimone, Tjan, & Treisman, 2007; Strasburger et al., 2011; van den Berg, Johnson, Martinez Anton, Schepers, & Cornelissen, 2012; Whitney & Levi, 2011). A key question for both of these classes of discrete-feature models is “What are the ‘textons,' the ‘atoms' of crowding?” In other words, what things are averaged? What things are detected before the loss of position information? Are the atoms unbound features, such as the orientation of each display item? Or bound features such as conjunctions of orientation and color? Or object identity, like letters?

The other approach to modeling crowding (and texture perception) essentially says that the primitives of crowding are not atoms at all, but rather stuff (Adelson, 2001). This other class of models operates on continuous features, such as the outputs of a cascade of filtering operations and nonlinearities. For example, a number of researchers have attempted to reason about the implications of a simple pooling model that averages continuous feature measurements over a pooling region (Levi & Carney, 2009; Manassi, Herzog, Sayim, & Herzog, 2012; Manassi, Sayim, & Herzog, 2013; Saarela, Sayim, Westheimer, & Herzog, 2009). However, this simple pooling model, conceived of as pooling within at most a handful of feature bands, has been disproven by a number of experiments (Kooi, Toet, Tripathy, & Levi, 1994; Levi & Carney, 2009; Levi, Klein, & Hariharan, 2002; Livne & Sagi, 2007; Malania, Herzog, & Westheimer, 2007; Manassi et al., 2012; Manassi et al., 2013; Nandy & Tjan, 2012; Sayim, Westheimer, & Herzog, 2010; van den Berg, Roerdink, & Cornelissen, 2007), and serves more as a straw man than as a real contender to model crowding. In contrast, our Texture Tiling Model (TTM) represents its inputs with a high dimensional set of local image statistics (stuff), known to be good for capturing texture appearance (Balas, 2006; Portilla & Simoncelli, 2000). This model measures correlations of the magnitude of responses of oriented V1-like wavelets across differences in orientation, neighboring positions, scale, and phase correlation across scale, as well as the marginal distribution of luminance and luminance autocorrelation (Rosenholtz, Huang, & Ehinger, 2012; Rosenholtz, Huang, Raj, Balas, & Ilie, 2012). A similar model, measuring the same set of statistics, has been put forward to describe early visual cortex (Freeman & Simoncelli, 2011; Freeman, Ziemba, Heeger, Simoncelli, & Movshon, 2013). We have previously shown that this model can predict the results of a number of crowding experiments (Balas et al., 2009), among other phenomena (Rosenholtz, Huang, Raj, et al., 2012).

**Figure 1:**
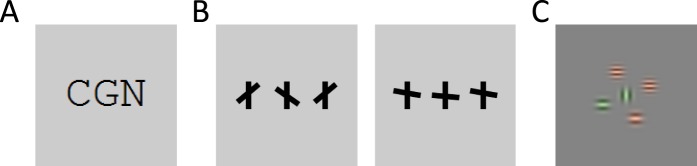
Example stimuli from three experiments discussed in this study. (A) Letter triplet from experiment 1 in Freeman et al. (2012). Observers viewed peripheral letter triplets and reported the identity of the central letter. (B) In Greenwood et al. (2012), observers reported the relative position and orientation of the central tilted crossbar. For example, the correct response for the first stimulus is “above the midpoint and counterclockwise,” while the correct answer for the second is “below the midpoint and clockwise.” In their experiment 4, Greenwood et al. (2012) varied the angular deviation of the crossbar from the horizontal in order to manipulate the overall probability of crowding. (C) Põder and Wagemans (2007) examined how crowding affects feature binding. The observer reported the color (red or green), relative spatial frequency (high or low), and orientation (horizontal or vertical) of the central Gabor. Each stimulus randomly had two, four, or six flanking Gabors. The background gray level was different in this experiment than the others.

In this paper, we examine the results from three different studies, each of which tested hypotheses about the supposed atoms of crowding—the discrete features upon which the mechanisms of crowding are presumed to act. One study on letter crowding (Figure 1A) indicates that the atoms are not individual letters (Freeman, Chakravarthi, & Pelli, 2012). A study with “T”-like stimuli (Figure 1B) suggests that the position and orientation of the crossbar are in some sense bound (Greenwood et al., 2012). A study with Gabor stimuli (Figure 1C) indicates imperfect binding between color, orientation, and spatial frequency (Põder & Wagemans, 2007). These experiments and their analyses are quite complex; given the differences between the stimuli, it is not obvious which discrete-feature model of crowding could explain the full set of results. On the other hand, a strength of image processing-based, continuous-feature models is their applicability to arbitrary stimuli. We use TTM as our candidate continuous feature model, since it has shown promise on a number of crowding results (Balas et al., 2009; Rosenholtz, Huang, Raj et al., 2012). We test whether TTM can account for the key results in all three studies. If so, this provides evidence in favor of continuous-feature pooling models of crowding, suggesting that perhaps the primitives of crowding are not atoms after all.

Testing this hypothesis is fairly complex. First, we must make TTM predictions for the previous experiments, essentially running our model through each experiment as a subject. In the next section, we describe the methodology for doing this, which involves using human observers. Next, we compare these TTM predictions to the original behavioral results. This alone, however, is insufficient. In each of the three papers described above, the authors fit computational models of various degrees of complexity in order to draw conclusions about the likely atoms of crowding; the authors' conclusions did not follow merely from eyeballing the data, nor from a few simple tests of significance. Here we ask, if TTM were a subject in the experiment, would the authors have come to the same conclusions by fitting its data (i.e., its predictions) as they did fitting their own experimental data? Taking this approach allows us to have a better sense of whether our model predicts key features of the data. Note that we are agnostic about both the details of the previous computational models and about the conclusions drawn from those results. We simply ask whether a crowding mechanism based on pooling of a rich set of continuous features could have led to the previously observed data and the resulting conclusions.

The purpose of this paper necessitates that we present a lot of information about the original experiments, the original models, and our attempts to test a unified account of these results. We have done our best to organize the paper to make it as clear as possible. First, we describe our standard methodology for generating predictions from TTM. Then, each original study appears in its own section, with four parts, to (a) present the motivation of the original study, (b) describe the experimental methodology, (c) compare our predictions to the original data, and (d) present the outcome of applying the computational modeling in the original study to the data of our model subject. Each section has a figure with sample stimuli and relevant results. Finally, we discuss the broader consequences of our findings.

## Generating predictions from TTM

To gather predictions of the TTM, we use the same methodology as in our previous work (Balas et al., 2009; Rosenholtz, Huang, & Ehinger, 2012; Rosenholtz, Huang, Raj et al., 2012; Rosenholtz, 2011; Zhang, Huang, Yigit-Elliott, & Rosenholtz, 2015). In short, the model represents a stimulus image as vectors of image statistics computed over localized, overlapping sections (pooling regions) of the image. These measured statistics are then fed into an algorithm that generates a new image that matches the original image's statistics, but is otherwise random. This methodology allows us to literally see the information encoded by the given set of statistics. The set of images that can be synthesized from a given image (with the same statistics but generated from different random seeds) form an equivalence class of the model. We call these synthesized stimuli “mongrels” (Balas et al., 2009).

The full version of TTM measures statistics over a number of pooling regions that overlap and tile the entire visual field. It takes hours to synthesize each image. Here we run the local version of the model, which extracts statistics from only a single pooling region. This reduces to Portilla and Simoncelli (P-S) texture analysis/synthesis (Portilla & Simoncelli, 2000; available at http://www.cns.nyu.edu/∼lcv/texture/), applied to stimuli we have blurred to account for reduced peripheral acuity. For stimuli such as those here, which lack larger scale grouping structure and are fairly homogeneous, syntheses of a single pooling region typically appear quite similar to those from the full version of the model.

For color stimuli, we use a color texture synthesis algorithm (available at http://www.cns.nyu.edu/pub/eero/colorTextureSynth.zip). This algorithm runs principle components analysis (PCA) on the original color image, and measures statistics on each of the three PCA channels. It synthesizes a new image by iteratively applying P-S texture synthesis to each of the three color bands, enforcing, on each iteration, the correlations between bands corresponding to the eigenvectors of the PCA. For grayscale images, this reduces to ordinary P-S.

We generate mongrels from image statistics measured on the stimuli in the three original studies. We generate many of these mongrels per condition and ask observers to perform essentially the same task as in the original study. The observers can view the mongrels for an unlimited time and without restriction of eye movements. Before each experiment, subjects were shown examples of the original stimuli along with their mongrels and told colloquially how the process of creating the mongrel can mix up locations, features, and legibility of the originals. This procedure attempts to minimize higher level factors in order to test the effect of low-level encoding on complex symbol recognition tasks (see Geisler & Chou, 1995; Zhang et al., 2015, for more discussion). Observer performance on the mongrel task gives us the predictions of TTM for a single pooling region.

The P-S texture analysis/synthesis method assumes that the image wraps around, meaning the top is adjacent to the bottom, and the left adjacent to the right (i.e., periodic boundary conditions). Essentially, it assumes that the image is on a torus. This leads to mongrels in which the “stuff” from the center of the original patch (e.g., the stimulus letters) can be centered on or near one of the edges, rather than the center of the image. This makes doing tasks like “report what letter appeared in the center of the original patch” somewhat tricky for naive subjects, even when told about the wraparound. As the center of the mongrel is completely arbitrary—it is simply a matter of what starting point one uses when unwrapping the torus—we correct for the wraparound by toroidally shifting each mongrel to move the center of mass of the foreground pixels to the center of the image.

## Experiments

### Can a simple substitution model account for crowding?

Substitution is undeniably a phenomenon in crowding, in which a subject, asked to report the identity of a target, instead reports one of its flankers. This phenomenon might suggest a mechanism of crowding, which measures features for target and flankers, but either does not encode their location or encodes it in a noisy way (Chung & Legge, 2009; Strasburger & Malania, 2013; van den Berg et al., 2012). This loss of location information would predict substitution of flankers for the target, though it is not the only mechanism that will do so (see Freeman et al., 2012, and Discussion).

Is crowding due to substitution operating at the level of entire objects like letters (i.e., does the mechanism of crowding identify complete letters, but lose their location?)? Freeman et al. (2012) examined the explanatory power of a simple substitution model at predicting responses in a crowded letter-identification experiment. In simple substitution, the response to a crowded stimulus is determined by independently sampling either the target or a flanker. This means that on a given trial, the response is either: the target, a letter similar to the target, a flanker, or a letter similar to that flanker. Importantly, because simple substitution is only a function of a single letter, any interaction between target and flankers, such as their similarity, does not directly influence the responses.

#### Their experiments

**Figure 2:**
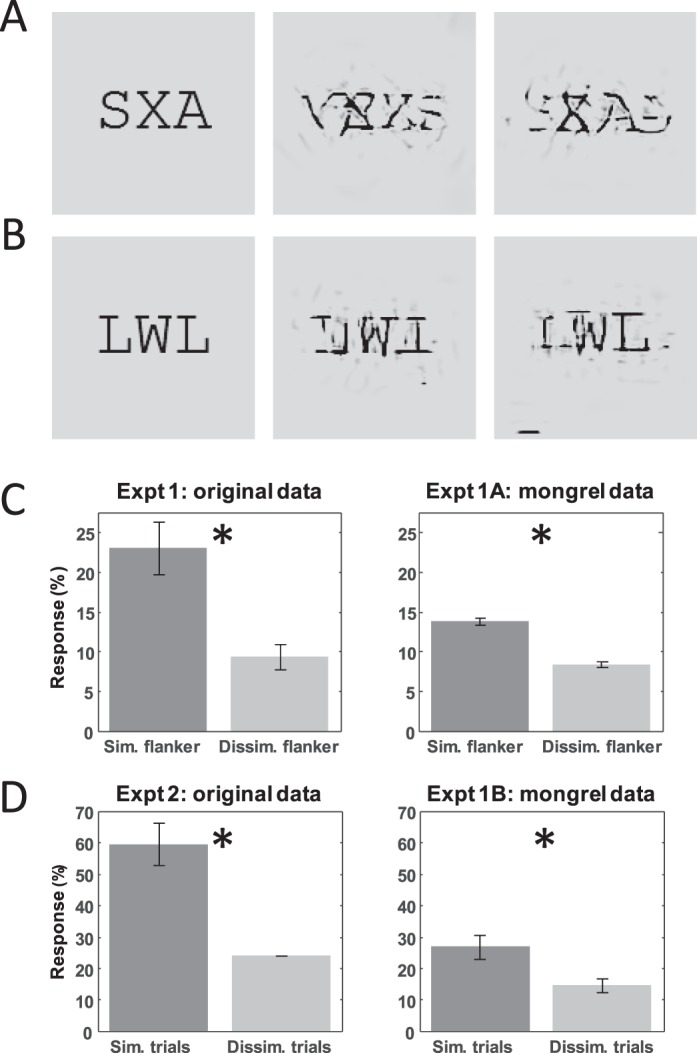
(A) Example stimulus from experiment 1 in Freeman et al. (2012), with two mongrels to its right used in our Experiment 1A. Mongrels are generated by iteratively enforcing image statistics on a random noise seed. Subjects were asked to report what three letters they believed the mongrel was generated from, free-viewing with no time limit. Notice that the mongrels show effects of substitution (swapping of letter positions), as well as complex interactions between letters. In agreement with Freeman et al. (2012), the flanker (A) more similar to the target (X) tends to be reproduced more faithfully in the mongrels than the dissimilar (S) flanker. (B) Original and mongrels for Freeman et al. (2012)'s experiment 2 (mongrels in our Experiment 1B). Interestingly, the letter “I” seems to appear in the mongrels, despite being absent in the original. (C) Subject reports of the similar flanker (first bar) compared to the dissimilar flanker (second bar), averaged across subjects, for both (left) Freeman et al. (2012) and (right) our Experiment 1A. Notice that in both studies, subjects reported the similar flanker significantly more often than the dissimilar (asterisk indicates *p* < 1 × 10^−5^; two-sided permutation test). (D) How often subjects mistakenly reported one of the identical flankers when they were similar (first bar) to or dissimilar (second bar) to the target, for both (left) Freeman et al. (2012)'s experiment 2 and (right) our Experiment 1B.

Freeman et al. (2012) presented subjects with triplets of letters that were viewed peripherally (Figure 2A, B). The subject's task was to report the identity of the central letter, the target, disregarding the flankers on either side. Freeman et al. (2012) based their conclusions largely on the results in their experiments 1 and 2, and so we address those two experiments here. In experiment 1, letter triplets were chosen such that one flanker was similar to the target and the other was dissimilar. Care was taken in choosing letter triplets to avoid introducing target–flanker correlations. This is necessary partially because observers more easily identify letters at the ends of a crowded array (Bouma, 1970). If target–flanker correlations were allowed, a subject who identified only the flankers could exploit the correlations to guess the target. A stimulus set was created for each subject such that each letter was used as a target, a similar flanker, and a dissimilar flanker. In experiment 2, an alphabet of two groups of three letters each (LTI, MNW) was used for all subjects. Within each group, the letters are similar, but between groups, they are dissimilar. The flankers were identical to each other in the second experiment, and both the target and flanker identities were chosen randomly on each trial.

Freeman et al. (2012) proposed that a key prediction of simple substitution is that subjects will report a similar flanker just as often as a dissimilar flanker. They found that this null hypothesis does not hold, and concluded that simple substitution cannot account for letter-identification performance under crowding. A recent paper (Hanus & Vul, 2013), however, argued that simple substitution with letter confusions could account for increased similar flanker reports, based on their own modeling of the impact of individual letter confusability. We remain largely agnostic to the specifics of the models discussed in these two studies, and focus on the phenomena of similar versus dissimilar flanker reports (see also Bernard & Chung, 2011).

#### Stimuli and procedure for generating TTM predictions

We tested five subjects in our reproduction of experiment 1 from Freeman et al. (2012). Four were experienced observers in previous mongrel experiments, and all were naive as to the design of the experiments. (Three were lab members, and thus may not have been completely naive as to the purpose of the experiments.) We tested four experienced psychophysical observers in Experiment 2. Two of these observers were naive to the purposes of the experiment, one of who also participated in Experiment 1. Of the two remaining observers, one was an author.

Using P-S synthesis, we generated mongrels of the letter triplet stimuli in (Freeman et al., 2012; Figure 2A, B). Prior to their first main experiment, Freeman et al. (2012) measured the confusion matrix for each observer between uncrowded peripheral letters with added noise. The confusion matrix was used to design a set of stimuli in experiment 1 for each observer, such that each letter in the alphabet was associated with a similar and dissimilar letter, resulting in a unique letter triplet of a target, a similar flanker, and a dissimilar flanker. Rather than carry out this procedure for each subject, we used the stimuli generated for their first observer (JF) for all observers in the mongrel task. Given the success of observer-independent letter-confusion modeling (Watson & Fitzhugh, 1989), it is reasonable to assume that letter-confusion matrices do not differ greatly in structure between observers, an assumption verified by Hanus and Vul (2013). We will show that we (similarly to Hanus & Vul, 2013) can nonetheless distinguish between simple substitution and pooling models. For each of the original 52 stimuli (each letter of the alphabet appears with equal frequency as the target, and the similar flanker can appear first or last in the triplet), we generated 10 unique mongrels (i.e., 10 members from the equivalence class of the original stimuli, according to TTM). This gave us a total of 520 trials per observer. Letter size and spacing were matched to the original experiment as closely as possible. Trial order was randomized for each subject.

To generate predictions for their second experiment, we generated letter triplets out of two groups of three letters, as in Freeman et al. (2012). Enumerating all possible combinations produces 36 unique triplets. We generated 10 mongrels for each triplet. Stimulus order was randomized for each subject.

In both experiments, instead of instructing the subjects to name a single target letter in the center of the mongrel image, we required subjects to enter three letters and used the second letter as the response. We did this to facilitate performing possible additional analyses of the responses (e.g., how errors in flanker reports relate to target errors; as such analyses are not relevant to the present paper, we do not present them here). For Experiment 1, subjects were informed that in the original patch, all letters occurred with equal frequency in all three locations, and that each mongrel represented a triplet of three unique letters. For Experiment 2, they were told the six possible letters (examples of which were shown on the screen at all times) and the flankers would always be identical. Response time was unlimited and subjects received no feedback.

#### Results

##### Data analysis:

In Experiment 1, trials in which subjects mistakenly reported too many letters, too few letters, or repeated letters were discarded (0.62% of all trials). Subjects reported the correct letter on 24.9% ± 1.1% (*M* ± *SE*) of trials, which is well above chance (1/26, or 3.85%), *p* < 1 × 10^−10^. This is noteworthy because unlike simple substitution, the TTM model does not explicitly encode information about letter identity; pooled image statistics are sufficient to support identification of letters. Subjects reported the similar flanker on 13.8% ± 0.4% of trials and the dissimilar flanker on 8.4% ± 1.6% of trials (Figure 2C). The two types of flankers are not reported equally (*p* < 1 × 10^−5^; two-sided permutation test), consistent with what Freeman et al. (2012) observed in their first experiment. This result was also significant for four of the five subjects individually (*p* < 0.035; *p* = 0.12 for the fifth; two-sided permutation test).

In Experiment 2, any trials in which subjects did not report identical flankers were discarded (1.39% of trials). Subjects reported flankers more often when they were similar (26.8% ± 3.8%) to the target than when they were dissimilar (14.4% ± 2.1%; Figure 2D). This trend is highly significant (*p* < 1 × 10^−5^; two-sided permutation test). This difference in flanker reports is qualitatively if not quantitatively similar to the results from Freeman et al. (2012), who found that flankers were reported 60% ± 6% of the time on similar-flanker trials and 24% ± 1% on dissimilar flanker trials. Subjects in the mongrel experiment had higher overall reports of absent letters than in the Freeman et al. (2012) study. In short, we find that in both Experiments 1 and 2, similar flankers are reported more often than to be expected by a pure substitution model, and thus our data support the notion that simple substitution is not a complete account of crowding.

##### Applying their models to TTM predictions:

Freeman et al. (2012) interpreted their first experiment as showing that simple flanker substitution (where responses are based on, but not necessarily identical to, a flanker chosen at random) cannot account for letter-similarity effects in crowding, which is consistent with our results. Moreover, using their mixture modeling technique, we determined that flanker substitution could at most account for 72% of responses in our mongrel experiment, compared to the 55% found by Freeman et al. (2012).

Similarly, using the mixture model from their experiment 2, we found simple substitution to account for at most 84% of trials. These results, taken together, suggest that substitution cannot be a full account of crowding. While our percentages attributable to substitution are higher than those found in the original study, it is important to keep in mind that the mixture model maximizes the role of substitution in fitting the data; only trials for which it is impossible for substitution to explain are attributed to pooling. We still reach the same conclusion about simple substitution; in other words, if our model were a subject in their experiment, they would have drawn qualitatively the same conclusions (if quantitatively different) as they did when looking at actual behavioral data.

### Do different visual features crowd in concert or independently?

If the atoms of crowding are not whole letters, as discussed in the previous subsection, what aspects of the stimuli are encoded prior to crowding (i.e., loss of location information or computation of summary statistics)? Greenwood et al. (2012) designed a set of experiments to determine which visual processing stage is responsible for crowding. One natural question to ask is whether different features of a stimulus, say position and orientation, are treated independently or bound together when crowding occurs. In a substitution-style model, in which some amount of recognition occurs (bars vs. corners vs. shapes and so on) prior to loss of position information, more binding implies that crowding operates at a later stage. Similarly, some models suggest that features of discrete items are identified and then averaged, producing crowding. The complexity of the feature measured and then averaged may suggest something about the stage at which crowding occurs. Thus, for both substitution and averaging classes of models, how much binding occurs gets at the important question of where in visual processing the mechanisms of crowding operate. One should note, however, that for models measuring image statistics rather than features of discrete items, the connection between amount of binding and stage of visual processing is not as straightforward. As we will see in our mongrels, a rich set of image statistics can capture a fair amount of information about feature binding even when that information is not explicitly encoded, much as the statistics capture some information about letter identity without explicit encoding of said identity.

Greenwood et al. (2012) fitted two models to their data. In both, crowding (i.e., averaging) mechanisms either operated on a given trial or they did not, in a way that depends probabilistically on the stimulus. In one model, this gating occurred for position and orientation features independently. In the other, whether or not orientation crowded also gated whether position crowded. They found that the latter model in which one gate controlled crowding for both features better fit the data. This result led them to the conclusion that binding of features occurs prior to the mechanisms of crowding. Here we compare the fits of their two models to our mongrel data and ask whether TTM would have led to the same conclusion.

#### Their experiment

**Figure 3:**
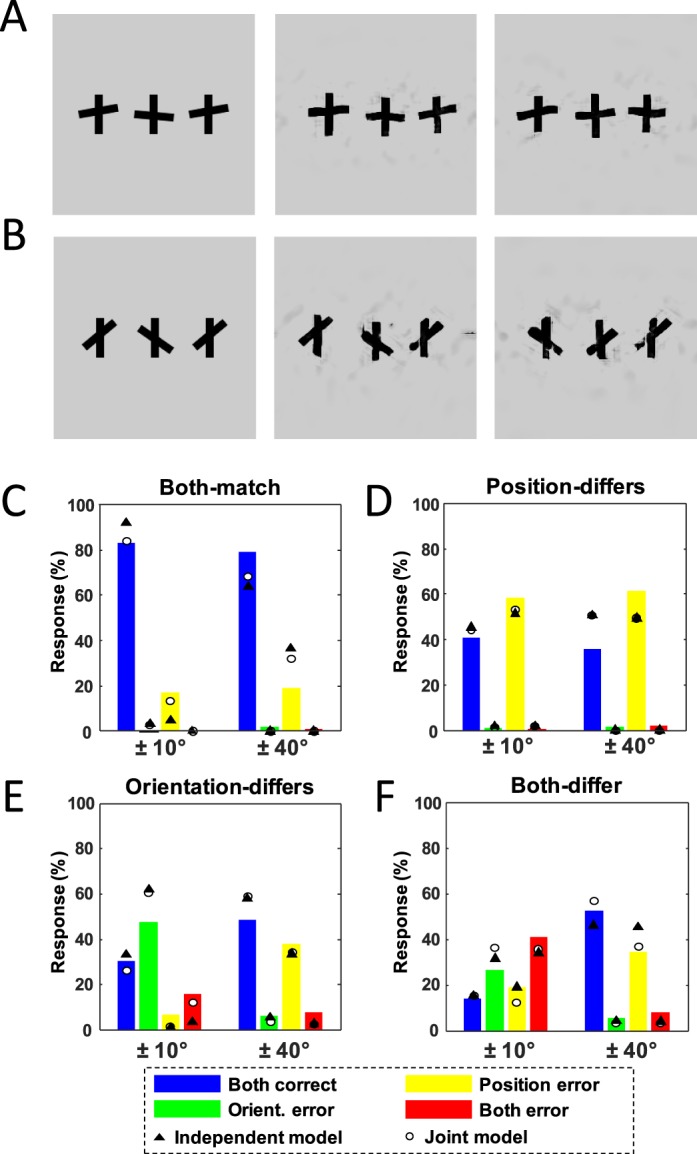
(A, left) An example stimulus from Greenwood et al. (2012) displaying strong crowding and a difference in both position and orientation between target and flankers (both-differ condition). Orientations of the target and flankers (target at 5**°**, flankers at −10**°**) are relatively close to zero. The correct response to the orientation and position (relative to the vertical bar's midpoint) of the center crossbar in this case would be “clockwise and below.” (A, right) Two of the corresponding mongrels. Notice that the orientation of the center object becomes ambiguous in the left mongrel and swaps with a flanker in the right mongrel. (B, left) Example from the weak crowding condition for which both position and orientation differ. Correct response to these stimuli would also be “clockwise and below.” The orientation difference between target and flankers is large here (target at 35**°**, flankers at −40**°**). (C–F) The average data over four subjects (bars) along with averaged model fits (circles and triangles). Each subplot corresponds to a particular relationship between target and flankers, just as in Greenwood et al. (2012). The different colors are different types of responses the subjects made. Within each plot, the bars are grouped according to the orientation of the target. Notice that subjects tend to make position errors when the target and flankers differ in position (D). They make orientation errors only when orientation is different (E) by a small amount (±10**°**, strong crowding) but not when orientation differs greatly (±40**°**, weak crowding). In the case where both position and orientation differ (F), subjects tend to make errors in both features under strong crowding. When crowding is weak, though, subjects get both features correct most often. The model fits in the last plot (F) show that the independent model (solid triangles) predicts more position errors than the data show. The joint model (white circles), however, correctly predicts a greater number of correct responses than position errors in this case.

Greenwood et al. (2012) presented triplets of cross-like symbols in subjects' periphery in their experiment 4 (Figure 3A, B). On each trial, one cross-like target was flanked by two other cross-like symbols, and subjects were to report the relative position and orientation of the target crossbar (above or below the midline? tilted clockwise or counterclockwise?). In some conditions, the orientation of the target and flanker were close to zero, thus inducing strong crowding (±10°; Figure 3A). In other conditions, it was very different from zero, thus inducing only weak crowding (±40°; Figure 3B). The strength of crowding as a function of orientation difference between target and flanker was determined separately in their experiment 1. By changing the level of orientation crowding independently of position crowding, Greenwood et al. (2012) argued that they could test whether crowding in one dimension induces it in the other, or whether they crowd independently. (In what follows, strong and weak crowding refer to experimental conditions, not performance.)

#### Stimuli and procedure for generating TTM predictions

We tested four subjects, all experienced psychophysical observers. All subjects had prior experience viewing mongrels. One was an author and one was naive to both the design and purpose of the experiments. The other two were lab members and naive as to the design but perhaps not to the broad purpose of the experiment. Because this task was quite unnatural and difficult, we used observers experienced with mongrels in order to focus on difficulty due only to information loss by the model. For each of the 32 original stimuli in experiment 4 from Greenwood et al. (2012), we generated 20 synthetic mongrel images (Figure 2A, B for example), for a total of 640 trials per subject. Subjects in the mongrel experiment were asked to do their best to infer from the appearance of the mongrel the orientation and position of the target crossbar in the original stimulus (from which the mongrel was derived). Subjects were shown examples of original stimuli and told how those original stimuli were generated (e.g., that the irrelevant flankers were always identical to each other). They were also told that when stimuli are synthesized, the objects can switch positions, flip, etc. Subjects had unlimited viewing time to respond to the orientation and position of the target crossbar by selecting one of four possible responses. To match the conditions in Greenwood et al. (2012), trials were split into blocks of 320 trials each. Each block had trials with either strong (difficult) or weak (easier) crowding. The order of blocks was counterbalanced between subjects, and subjects received no feedback.

#### Results

##### Data analysis:

As in Greenwood et al. (2012), we separate the data into four categories that correspond to the relationship between the target and flankers: both-match, position-differs, orientation-differs, and both-differ (Figure 3C through F). In each of these categories, when crowding occurs (leading to weighted averaging a la Greenwood et al., 2012), the observer will be more likely to report the value of a flanker's features. According to this model, in the both-match condition, averaging target and flanker responses would still lead to a correct response (e.g., the average of three positions above the midline is above the midline). We find that our subjects are most likely to be correct in both features, regardless of crowding level (Figure 3C). In the position-differs case, the predominant responses in our data are position errors (also regardless of crowding level), although not much more frequent than correct responses. This suggests that while position judgments were crowded, if gating occurred a la Greenwood et al. (2012), it would operate on around or slightly less than 50% of trials (Figure 3D). In the orientation-differs case, the results are more complex. In the strong crowding subcase, observers most commonly responded with an orientation error, and to a lesser extent (but not significantly less), they responded correctly, suggesting 50% or more of trials were crowded in orientation. In the weak crowding case, however, orientation errors dropped to nearly zero, and the most common response was to be correct (Figure 3E). This is not surprising, as Greenwood et al. (2012) designed the weak crowding condition with large tilts to preclude orientation errors.

Importantly, according to Greenwood et al. (2012), the key set of trials consists of those in which both the position and orientation differ between target and flankers (both-differ; Figure 3F). In the case of strong crowding, their subjects most commonly made mistakes in both position and orientation. When crowding is released in the weak crowding condition, however, the dominant response in their experiment was to be correct in both position and orientation. This effect is also prominent in our results.

To be specific, when crowding is released by increasing the difference in orientation between target and flankers, subjects have fewer orientation and position errors. This can be shown by computing the proportion of correct responses expected if releasing crowding only affected orientation judgments, and comparing it to the actual proportion of correct responses. If releasing crowding only affected orientation judgments, the proportion of correct responses in the weak crowding case should be equal to the number of correct responses with strong crowding, plus the number of orientation errors with strong crowding, minus the number of orientation errors with weak crowding. If the actual number is higher, then releasing crowding in orientation also releases crowding in position, supporting Greenwood et al. (2012)'s joint model of crowding. If the expected and actual proportions are the same, then an independent model of crowding, where position and orientation don't interact, cannot be ruled out. We find that in three of four subjects individually, the proportion of correct responses is 22.5% ± 3.8% higher than expected if weak orientation crowding only affected orientation judgments (*p* < 0.025; two-sided permutation test for each subject), and higher (6.25%) but not significant for the fourth subject (*p* = 0.4581; two-sided permutation test for one subject). This is the same trend observed by Greenwood et al. (2012).

##### Applying their models to TTM predictions:

In order to interpret their results, Greenwood et al. (2012) fitted two models to their data. The models were similar in that both assume (a) noisy encoding of position and orientation, (b) a mechanism that gates orientation crowding on or off with a probability that depends on how close the target and flanker orientations are, and (c) the effect of crowding is modeled as a mechanism that takes a weighted average of the target and flanker features. The models differ in that one model (the independent model) has a separate parameter for the probability gating on or off position crowding, while the other (the joint model) posits that crowding in position happens if and only if crowding in orientation happens on a given trial. For a schematic of these models, see figure 6A in Greenwood et al. (2012). Greenwood et al. (2012) argued that if the joint model does similarly (given that it has one fewer free parameter) or better in explaining the data, this implies that crowding in position is tied to crowding in orientation (i.e., that crowding happens after the binding of position and orientation).

We fit these two models to our mongrel data and compare the fits. Because we had difficulty converging to a good fit using the models and data from Greenwood et al. (2012), we made one minor change in both of the models. Specifically, we used the thresholds determined from their experiment 3 to set the standard deviation of position noise in their model (their equation A4). This was done to keep parameter estimates at intermediate values and to help the error minimization converge more quickly. Importantly, this component of the modeling is identical for both models, and occurs before the binding step, so it should not affect the ability to distinguish the models.

We fit both models to each of our subjects individually and found that for each subject, the independent model (i.e., the model in which crowding happens independently for orientation and position) fit worse than the joint model. The root mean squared error of the independent model was 1.71 ± 1.1 percentage points worse than the joint model. The joint model fits the mongrel data better for each subject individually and overall. Compare this to the fits in Greenwood et al. (2012), where they found root mean square errors of 6.29 for the independent model, and 3.78 for the joint model, a difference of 2.51 ± 0.0012. The joint model also fits their data better. Given the difference between the fits of the independent and joint models, our data also supports the conclusion that orientation and position are bound prior to crowding.

### How does crowding affect feature integration?

Põder and Wagemans (2007) also studied to what degree feature binding survives crowding. They asked whether fully bound items are mixed up by crowding (high-level substitution), whether the mixing happens in each feature band separately, or something in between. By analyzing feature errors and fitting models to the subject responses, they concluded that the level at which crowding occurs is neither extreme, but includes partially bound features. For example, the authors found a correlation between the number of flankers with a particular feature value and the number of times that feature value was erroneously reported. This correlation, however, was not perfect, suggesting some binding of the target features. Additionally, they argued that a Feature Integration Theory (FIT)-style model in which features bind probabilistically within a spotlight of attention fits the data very well, despite having only two free parameters.

We used the method described by Põder and Wagemans (2007) to generate stimuli, which we then used to generate mongrels. Subjects viewed the mongrels and did the same task as in the original experiment. As in the previous sections, we use the same analysis and modeling on our data to test whether we would arrive at the same conclusions about feature binding in crowding.

#### Their experiment

**Figure 4:**
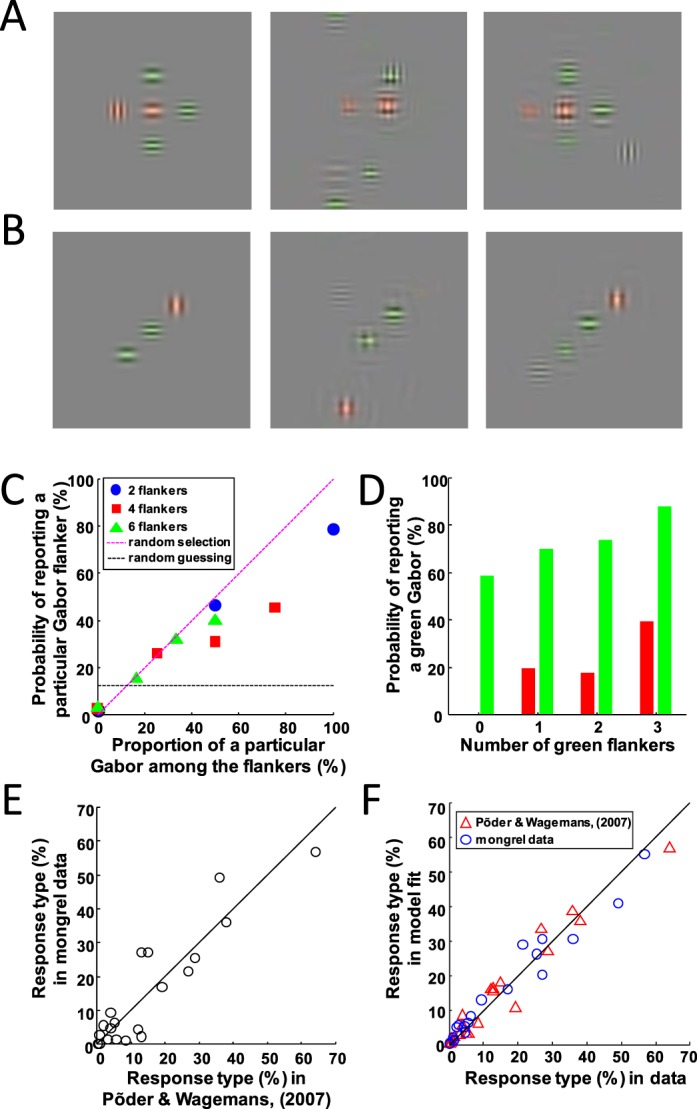
(A) Example stimulus from Põder and Wagemans (2007), consisting of a central Gabor target with four flankers, along with two mongrels to its right. Notice that in the mongrels, fully bound objects (e.g., colored Gabors) appear, but their component features and relative positions may change. The first mongrel seems to have an illusory conjunction, a green vertical Gabor; there were none in the original stimulus. The second mongrel shows that TTM's representation can encode some information about the overall spatial arrangement. (B) Original and mongrels of a two-flanker stimulus. Notice that the first mongrel also shows signs of an illusory green vertical, whereas the second mongrel shows swapping of whole objects. (C) Results of Experiment 3. All results in this figure are from data pooled over all subjects. Probability of mistakenly reporting a particular flanker as a function of the proportion of times that flanker appears in the stimulus, computed over all subjects' data. Predictions from random selection and random guessing are overlaid. Responses fall between random selection and random guessing, as in Põder and Wagemans (2007). (D) Probability of reporting green in Experiment 3 as a function of the number of green flankers. Green bars indicate a green target, while red bars indicate a red target (and thus incorrect trials). All data come from trials with four flankers. Features that appear more often are reported more often, again in agreement with Põder and Wagemans (2007). (E) Plot of performance in Põder and Wagemans (2007), compared to performance in our Experiment 3. Each point's *x*-value corresponds to a bar in figure 7 of Põder and Wagemans (2007), where they organize the data by number of flankers in the stimulus and the number of feature errors in the response. (F) FIT-style model fits to data from Põder and Wagemans (2007; red triangles) and our Experiment 3 (blue circles). Notice that both data are well fit by the model, which only has two free parameters.

Using Gabor patches as stimuli, Põder and Wagemans (2007) tested the effect of flankers on an identification task in which the observer must report three features of the target. Each Gabor patch had three orthogonal properties: color (red or green), orientation (vertical or horizontal), and spatial frequency (high or low). The target was selected randomly from the eight possibilities (2 × 2 × 2). The target was flanked by two, four, or six Gabors, randomly chosen on each trial. The flankers' features were randomly chosen such that no flanker was identical to the target. The flankers were placed around the target, with their centers regularly spaced on a notional circle of radius 0.8° (Figure 4A, B). On each trial, the array of Gabors was briefly displayed peripherally, and subjects indicated the target identity by selecting one of eight possible responses.

#### Stimuli and procedure for generating TTM predictions

Four subjects participated in our experiment. One was an author, two had significant experience with mongrels, and the fourth had a small amount of experience with mongrels. All subjects except the author were naive to the purposes of the experiment. All properties of the stimuli were chosen to match those of the original study. The subjects were instructed to respond to what they thought were likely the features of the central target in the original stimulus. Subjects responded by choosing one Gabor patch of the eight possibilities displayed at the bottom of the screen and received feedback on each trial.

#### Results

##### Data analysis:

In their analysis, Põder and Wagemans (2007) organized the data in several different ways to make sense of the phenomena. First, they noted that for all subjects, performance decreases with increasing number of distractors. They also plotted the predictions of a random selection model, where the subject randomly reports one of the Gabors in a given stimulus (essentially losing all position information). This random selection model predicts the same trend but severely underestimates actual performance. Our data (for individual subjects and on average) also show both this decline in performance with increasing number of distractors and better performance than predicted by random selection. This means that while TTM's representation loses information about the stimulus, it does preserve some spatial configuration information. One can directly observe this in the mongrels (Figure 4A).

Another important finding reported by Põder and Wagemans (2007) is that subjects' probability of responding with a particular distractor is proportional to the prevalence of that particular distractor in a given display. They attempted to discriminate between two mechanisms that might drive this performance: random selection (mentioned previously) or misbinding, where the subject reports a combination of features randomly chosen from those present in the stimulus. They showed that the probability of reporting a flanker is proportional to how prevalent that flanker is in a particular stimulus. However, the proportionality is less than 1, indicating that at least some responses are not due to random selection. Likewise, they provided evidence for misbinding by showing that increasing the number of times a particular feature value appears among the flankers increases the likelihood of it being reported. For example, having more green flankers in the display leads to a higher probability of subjects reporting a green target. We find strong evidence that TTM produces both of these effects (Figure 4C, D).

##### Applying their models to TTM predictions:

Põder and Wagemans (2007) presented several computational models of their experiment. The one they found most compelling is a FIT-style model. Briefly, this model asserts that responses on a given trial are a result of a doubly stochastic process: First, a “center of attention” is chosen randomly from a two-dimensional normal distribution centered at the stimulus center (the target). The variance of this normal distribution—essentially the error in centering attention on the target—is a free parameter of the model. Second, the probability of choosing features from a given display item falls off with distance from the center of attention, according to another two-dimensional normal distribution whose variance is a second free parameter of the model. Põder and Wagemans (2007) simulated this model and found the values of the free parameters that best fit the data. Specifically, they fitted the distribution of responses as a function of the number of flankers, the number of feature errors (how many features differed between the response and the target), and whether the response was present in the flankers (see figure 7 in Põder & Wagemans, 2007). Organizing the data in this way, we compare the results of our study and theirs in Figure 4E. They found that this model fits well compared to other models tested (Põder & Wagemans, 2007). They found the best-fitting variance parameters for both normal distributions to be 0.32° visual angle. We also fit their model to our data and found it to fit well, with the optimal parameters being effectively 0.48° and 0.2° of visual angle in the original crowding experiment (Põder & Wagemans, 2007). The model fits to their and our data are shown in Figure 4F.

## Discussion

The TTM of visual processing, in which a rich set of image statistics are measured over sparse pooling regions that tile the visual field, accounts for a variety of crowding phenomena (Balas et al., 2009; Rosenholtz, Huang, & Ehinger, 2012; Rosenholtz, Huang, Raj, et al., 2012; Zhang et al., 2015). Importantly, because the model operates on images rather than hand-labeled, experiment-specific features, the model is flexible enough to make predictions for arbitrary stimuli. We have shown here that three reasonably different crowding phenomena can be captured by this one model.

First, Freeman et al. (2012) found that when viewing letter arrays in the periphery, performance cannot be described by independent position and identity confusion; a more complex mechanism (pooling) is required to account for crowding. We also find our model predicts that their crowding results cannot be completely explained by simple substitution.

Second, when we compare the predictions of TTM to those of Greenwood et al. (2012)'s model, we find that both result in the so-called “release of crowding” for position under conditions of weak crowding for orientation; orientation and position seem to be in some sense bound by peripheral vision. It is important to note that TTM predicts this release from crowding phenomenon, even though it has no explicit gating mechanism that turns crowding mechanisms on or off depending upon the stimulus. This suggests that given the information encoded and lost by peripheral vision, some stimuli inherently lead to more or less difficult recognition of the target.

Finally, in Põder and Wagemans (2007), subjects attempted to identify peripheral objects in which target and flankers varied independently along three orthogonal feature dimensions. Responses seemed to be a mix of substitution of whole flankers (full binding), and substitution of partially bound features (including illusory conjunctions). They found that a parsimonious explanation of this phenomenon was a FIT-style model with a soft-edged and semirandomly placed attentional window. The TTM also predicts a mixture of whole flanker and illusory conjunction reports and importantly generates data that are well approximated by the same FIT-style model.

Predictions made by the TTM, overall, are thus consistent with a wide range of results from a diverse set of experiments. The qualitative success of the model is encouraging. This success likely derives in part from it belonging to a class of models that includes a cascade of oriented bandpass filtering, nonlinearities, and pooling over sizeable but sparse regions in order to compute a large number of summary statistics. The specific image statistics hypothesized by the model may not be exactly right, as also suggested by the present work. Though the qualitative results are good, quantitatively, our model predictions did not always agree with the data. For example, in the letter-identification task (originally from Freeman et al., 2012) subjects viewing mongrels in our study reported significantly more absent letters than in the original study (Figure 2C; the decrease in “similar flanker” responses in our data as compared to Freeman et al., 2012, is attributable to absent-letter responses). The number of reports of absent letters might decrease with the use of the full version of TTM, which gathers additional information from multiple pooling regions. However, in addition, the model statistics may need further refinement. We would have been surprised if the image statistics in Portilla and Simoncelli (2000), developed to capture texture appearance, turned out to be precisely the statistics necessary to predict the phenomena of visual crowding.

**Figure 5:**
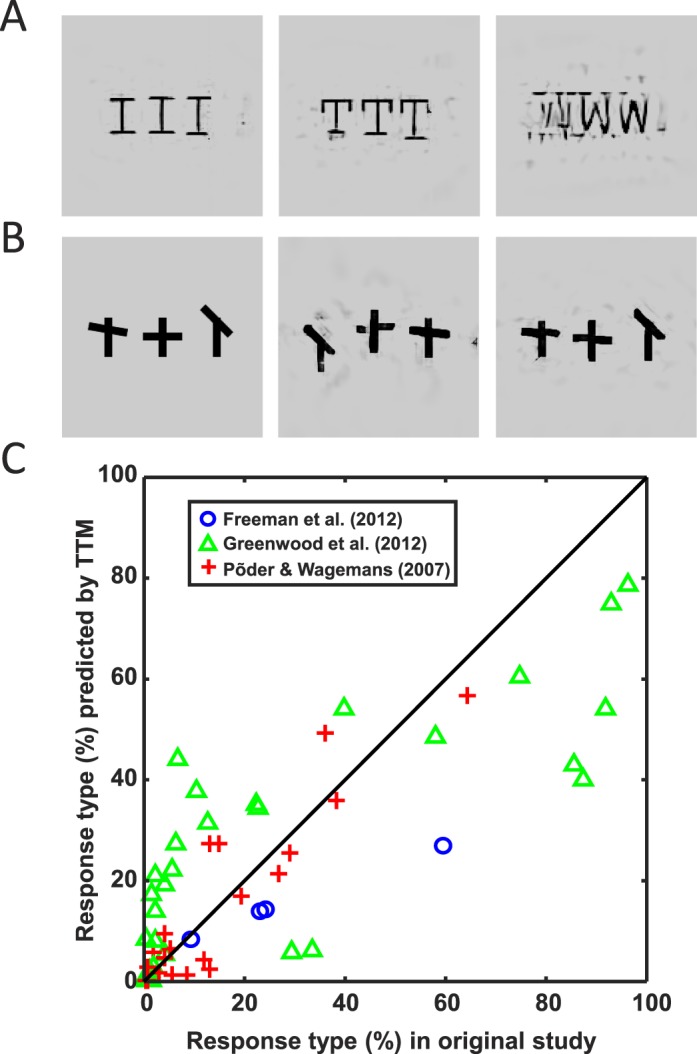
(A) Three mongrels of letter triplet stimuli from Freeman et al. (2012) in which the letters in the original stimulus were identical (III, TTT, and WWW). Notice that identical letter triplets tend to be better represented by the mongrels than when the letters are different (Figure 2A, B), although they aren't perfect (in the WWW case for example). The improvement in representation comes from the fact that identical letter triplets form a more coherent visual texture, irrespective of the letter identity itself, and textures in general are better represented by summary statistics. (B) Example stimulus that is similar to Greenwood et al. (2012)'s stimuli, and two mongrels to its right. Notice that in the mongrels, orientation and position are bound; the tilted bar intersects the vertical bar in each object at the same place as the original, even though objects can become shuffled around. (C) Plot of all relevant data from the original three studies (horizontal axis) versus TTM predictions (i.e., the data in our three experiments; vertical axis). The data points for Freeman et al. (2012) derive from Figure 2C, D. The data points from Greenwood et al. (2012) are those in Figure 3C through F. The data points from Põder and Wagemans (2007) are copied from Figure 4E. A viable model of crowding must be able to generate a plot like this one, where predictions can be generated for arbitrary stimuli in the form of images.

One powerful feature of TTM is the ability to visualize the equivalence classes of the model, as represented by the mongrels. Mongrels can be used to gain intuitions about peripheral vision, without having to run an entire experiment. These intuitions facilitate the development of new experiments and theories. For example, we have observed that triplets of identical letters (e.g., AAA) were better preserved in mongrels than triplets of nonidentical letters (e.g., ABA; Figure 5A vs. Figure 2A, B, respectively). The hypothesized encoding better represents identical letters, likely leading to easier recognition in such cases. This is in addition to any decision-making effects, in which identical letters also have an advantage because reporting a flanker also gives the correct answer. An interesting effect that becomes apparent when viewing mongrels of letter triplets is that letter order also makes a difference; mongrels suggest that GNW, for example, may result in a qualitatively different percept than WNG. This makes sense, as the local correlations measured by the model will be different in the two cases. Future experiments might determine whether or not letter order actually makes a difference in letter triplets viewed peripherally.

The mongrels we generated of the stimuli in Põder and Wagemans (2007) resulted in illusory conjunctions mainly between color and other features. In other words, spatial frequency and orientation of Gabors were more tightly bound to each other than color was to either of the other features. A follow-up experiment could more carefully examine the nature of the illusory conjunctions in the crowded periphery. Rosenholtz, Huang, Raj et al. (2012) previously demonstrated that TTM predicts orientation–color illusory conjunctions underlying difficult conjunction search tasks.

An interesting observation from the mongrels is that the locations of individual objects are often jumbled up such that the target does not necessarily appear in the center of the array. In other words, the model predicts substitution-like phenomena without a substitution-like mechanism. At least for some simple stimuli, the image statistics measured by TTM are sufficient to encode the correct binding between features of the same object. This means that if the original stimulus was the letter triplet ABA, the mongrels would often contain two As and one B. If the subject knows that the stimulus will contain two of the same letter and one different, they can bring to bear this knowledge and pick the target by choosing the letter that is different. Rather than a crowded letter identification task, as the experimenter may have intended, the subject may actually be doing an oddball identification task. By better understanding the representation stage, as visualized by the mongrels, we can better understand the role of decision-making in performing tasks under conditions of crowding. Experimenters must not only model the encoding losses due to crowding, but also take into consideration the subject's prior knowledge of the task.

This study sets a new benchmark for crowding research by attempting to predict a range of experimental results with a single model (Figure 5C). Looking at Figure 5C, a standard linear regression model results in a best-fit line with slope 0.608 ± 0.057, intercept 0.07 ± 0.02, and adjusted *R*^2^ = 0.665. (Putting aside, for purposes of discussion, that this standard fitting procedure is not strictly correct for these data. Some of the data points represent percentages of trials with a given response, which must add up to 100%; this introduces unavoidable correlations between the values within the same study. Furthermore, the percentage data theoretically violate assumptions of homoscedasticity [uniform variance].) If we assume TTM is a complete model of peripheral encoding, without any fitted parameters, then the line fit to the data should have slope 1 and intercept 0. Enforcing this results in *R*^2^ = 0.39, with no free parameters. One could add a single fitted parameter (e.g., a multiplicative constant on the mongrel predictions) in order to improve the fit, but the value of that parameter would not be easily interpretable in the context of understanding the model. There is clearly room for improving the model in future studies.

Nevertheless, TTM makes testable predictions without having to hand-tune it to the task-relevant stimulus features or augment it with ad hoc crowding mechanisms like grouping or gating. This is a direct result of the visual texture representation it uses, which is image-computable and operates on stuff rather than presegmented and processed things like items and bars. Given the parallels between models of texture processing and models of crowding, it is worth noting that stuff models of texture perception have been more successful than thing models (Rosenholtz, 2014). Models based on image statistics make testable predictions for a far broader range of input stimuli and have performed well across a range of stimuli and tasks (Heeger & Bergen, 1995; Malik & Perona, 1990; Portilla & Simoncelli, 2000; Rosenholtz, Huang, Raj et al., 2012; Zhang et al., 2015). In order to be viable, future models of crowding must, like TTM, be image-computable, and able to make predictions on the wide range of stimuli behavioral researchers can throw at them.

## Supplementary Material



## Footnotes

1 Also referred to as the foveola, or rod-free fovea.
