# Supplementary figures and images for: Pooling of continuous features provides a unifying account of crowding
Source: J Vis. 2016 Feb 26;16(3):39. doi: 10.1167/16.3.39 (PMC4790193; doi:10.1167/16.3.39)

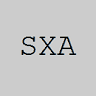

Supplement: Supplementary file 1 [file i1534-7362-16-3-39-icon.gif]
